# The complete mitochondrial genome of *Heteropriacanthus cruentatus* and implication of phylogenetic status

**DOI:** 10.1080/23802359.2021.1950062

**Published:** 2021-07-19

**Authors:** Peng Sun, Xingwei Yuan, Yazhou Jiang, Jianzhong Ling

**Affiliations:** Key Laboratory of East China Sea Fishery Resources Exploitation, Ministry of Agriculture, East China Sea Fisheries Research Institute, Chinese Academy of Fishery Sciences, Shanghai, China

**Keywords:** *Heteropriacanthus cruentatus;* mitochondrial genome, phylogenetic analysis, Priacanthidae

## Abstract

The complete mitochondrial genome of *Heteropriacanthus cruentatus* has been obtained and annotated through Illumina next-generation sequencing. This mitogenome was found to be 16,506 bp in length, containing 13 protein-coding genes (PCGs), 22 transfer RNA genes (tRNA), and 2 ribosomal RNA genes (rRNA). This overall base composition of the complete mitogenome for this species included 27.52% A, 24.46% T, 16.99% G and 31.04% C. The phylogenetic analysis revealed that the *H. cruentatus* has the closest relationship with *Pristigenys niphonia*. This study provides an important resource for reviewing the phylogenetic relationships and taxonomic status of this species.

The family Pricanthidae is distributed in tropical and subtropical seas around the world near coral reefs or rock formations, which consists of 4 genera and 18 species of marine fishes known as bigeyes (Fernandez-Silva and Ho 2017). *Heteropriacanthus cruentatus* (Lacepède 1801) belongs to the family Pricanthidae and genus *Heteropriacanthus*, it has a wide distribution in Atlantic and Indo-Pacific oceans. This species is common in lagoons and seaward reefs with a usual depth between 3 and 35 m (Gasparini and Floeter 2001). *Heteropriacanthus cruentatus* was first used by Fitch and Crooke (1984), and also has some synonymous name such as *Priacanthus carolinus*, *P. cruentatus*, and *P. boops* (Gaither et al. 2015). In this study, we reported the complete mitochondrial DNA sequence information of *H. cruentatus* and evaluated the relationship in comparison with closely related species.

The *H. cruentatus* samples were collected from the central coastal area of Zhejiang in East China Sea (27.0170°N, 123.0330°E). Sample of muscle tissue was collected from a specimen deposited at the East China Sea Fisheries Research Institute, Chinese Academy of Fishery Sciences (Peng Sun, sunpeng0512@hotmail.com) under a voucher number HCR2020801002. The total genomic DNA was extracted from a piece of muscle tissue using the DNA Extraction Kit (Tiangen, Beijing), and the extracted genomic DNA was amplified and sequenced through llumina NovaSeq platform using paired-end (2 × 150bp) sequencing mode by Shanghai Personal Biotechnology Co., Ltd. Then, the whole genome DNA was assembled by the SPAdes (Bankevich et al. 2012) and annotated by the MITOS (http://mitos.bioinf.uni-leipzig.de/) (Bernt et al. 2013).

The complete mitochondrial genome of *H. cruentatus* is 16,498 base pair (bp) in length (GenBank accession number: MZ042267) including 13 protein-coding genes, 2 ribosomal RNA (12S rRNA and 16S rRNA) and 22 transfer RNAs. Its base composition is 27.52% A, 24.46% T, 16.99% G and 31.04% C. Most of these protein-coding genes start translation with initiation codon of ATG, except for cox1 (ATC) and nd6 (ATT). The termination codons were TAA for genes of *nd*1, *nd*2, *nd*4, *nd*5, *nd*6 *nd*41, *cox*1, *cox*2, *cox* 3, *atp*6, *atp*8, *cob*, and TAG for genes of *nd*3. The rRNA position from 69 to 1016 with a length of 948 bp (*rrn*S), and from 1143 to 2789 with a length of 1647 bp (*rrn*L). The two non-coding regions are comprised of a light strand replication origin (OL) and D-loop region. The OL with a 33 bp in length is locate between *trn*N (gtt) and *trn*C (gac). The D-loop region (506 bp) is located between *trn*P (tgg) and *trn*F (gaa).

Fifteen mitochondrial genome sequences from Lutjanidae, Oplegnathidae, Sparidae, and Pricanthidae of Priacanthiformes and Eupercaria were used for phylogenetic construction by MEGA 7.0 with maximum likelihood method (Figure 1) and bootstrap value of 1000 replicates (Sudhir et al. 2016). Results showed that *H. cruentatus* has a closest phylogenetic relationship with *Pristigenys niphonia*. This study will provide useful genetic information to the genetic evolution and taxonomic study for *H. cruentatus*.

**Figure 1.**
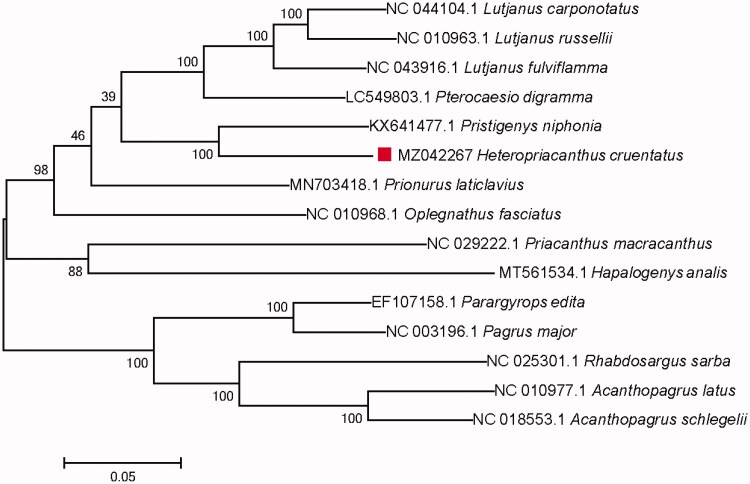
Maximum likelihood phylogenetic tree based on 15 complete mitochondrial genomes.

## Data Availability

The genome sequence data that support the findings of this study are openly available in GenBank of NCBI (https://www.ncbi.nlm.nih.gov/) under the accession number MZ042267. The associated BioProject, SRA, and BioSample numbers are PRJNA724929, SRR14663579 and SAMN18865290, respectively.
